# Should non-invasive prenatal testing be recommended for patients who achieve pregnancy with PGT?

**DOI:** 10.1186/s12884-024-06284-7

**Published:** 2024-02-01

**Authors:** Yunhao Liang, Meiyi Li, Jia Fei, Zhiheng Chen

**Affiliations:** 1 Center of Reproductive Medicine, Guangzhou Women and Children’s Medical Center, Guangzhou Medical University, Guangzhou, Guangdong, CN China; 2 Peking Jabrehoo Med Tech Co., Ltd, Beijing, CN China

**Keywords:** Preimplantation genetic testing, Non-invasive prenatal testing, Clinical outcomes

## Abstract

**Objective:**

To determine whether non-invasive prenatal testing is an alternative testing option to preimplantation genetic testing (PGT) in pregnant patients.

**Methods:**

This was a retrospective study of the clinical outcomes of patients who underwent PGT and invasive or non-invasive pregnancy testing after euploid blastocyst transfer at our IVF centre between January 2017 and December 2022.

**Results:**

In total, 321 patients were enrolled in this study, 138 (43.0%) received invasive pregnancy testing, and 183 (57.0%) patients underwent non-invasive testing. The mean age of the patients in Group 2 was higher than that of the patients in Group 1 (35.64 ± 4.74 vs. 31.04 ± 4.15 years, *P* < 0.001). The basal LH and AMH levels were higher in Group 1 than in Group 2 (4.30 ± 2.68 vs. 3.40 ± 1.88, *P* = 0.003; 5.55 ± 11.22 vs. 4.09 ± 3.55, *P* = 0.012), but the clinical outcomes were not significantly different. Furthermore, the clinical outcomes of patients undergoing invasive testing were similar to those of patients undergoing non-invasive testing with the same PGT indication.

**Conclusion:**

Our results suggest that non-invasive pregnancy testing is a suitable alternative option for detecting the foetal chromosomal status in a PGT cycle. However, the usefulness of non-invasive testing in PGT-M patients is still limited.

## Introduction

PGT is commonly used in artificial reproductive technology (ART) treatment [1–3]. It has been demonstrated that selecting euploid or no-gene defect embryos for implantation by PGT could decrease the chance of having a foetus with a chromosomal or genetic disease [4, 5]. PGT can be divided into 3 groups according to clinical indications: PGT for aneuploidy (PGT-A), PGT for structural rearrangement (PGT-SR) and PGT for monogenic conditions (PGT-M) [6, 7]. The clinical indications for PGT-A include advanced maternal age (≥ 38 years), recurrent abortion and recurrent implantation failure [8]. PGT-SR is used to detect chromosome structure rearrangements, including reciprocal translocations, the Robertsonian translocation, inversions, insertions, deletions and repetitions [9]. PGT-M is used to detect monogenic disease [10]. On the other hand, PGT-A is often combined with PGT-M to detect embryo aneuploidy [11, 12].

Although PGT can improve the pregnancy outcome of ART, it is not perfect. Victor et al. demonstrated that the aneuploidy concordance between trophectoderm (TE) biopsy and blastocyst biopsy is 93% [13]. Ou et al. obtained similar results: the aneuploidy concordance between trophectoderm biopsy and blastocyst biopsy was 70% [14]. The accuracy of PGT based on TE biopsy is related to embryonic mosaicism [15]. Mosaic embryos harbour both euploid and aneuploid cells, and the underlying mechanism is still unclear; therefore, false negative or positive results cannot be avoided [16, 17]. For this reason, patients who undergo PGT and who achieve pregnancy after implantation are required to undergo prenatal diagnosis [18, 19]. Invasive pregnancy tests (IPTs), including chorionic villus sampling (CVS) or amniocentesis, are required for the patients above, and the miscarriage risk prior to 24 weeks in patients who undergo CVS or amniocentesis is 1.39% and 0.91%, respectively; both of these rates are higher than those in the control group (1.23% and 0.58%, respectively) [20]. Therefore, procedure-related miscarriage risk is a concern, and this is the main reason why patients decline CVS or amniocentesis [21].

The discovery of cell-free foetal DNA in maternal plasma has enabled the development of non-invasive prenatal testing (NIPT) for the detection of foetal genetic status. It provides a safe, rapid, and high-efficiency approach for first-trimester aneuploidy screening [22]. A meta-analysis performed by Mackie et al. revealed that the detection sensitivity and specificity are respectively 0.989 and 0.996 for foetal sex, 0.993 and 0.984 for rhesus D, 0.994 and 0.999 for trisomy 21, 0.977 and 0.999 for trisomy 18, 0.929 and 0.999 for monosomy X, and 0.906 and 1.0 for trisomy 13 [23]. Phillips’ study revealed that the sensitivity of NIPT was 99.3% (95% CI 98.9–99.6%) for trisomy 21, 97.4% (95.8–98.4%) for trisomy 18, and 97.4% (86.1–99.6%) for trisomy 13. The specificity for each of the three trisomy types was 99.9% (99.9–100%) [24]. Zheng’s study obtained similar results to those of the studies mentioned above. The sensitivities of NIPT for trisomy 21, trisomy 18, trisomy 13, sex chromosome abnormalities, other chromosomal aneuploidies, and CNVs were all 100%, and the specificities were 99.90%, 99.94%, 99.96%, 99.82%, 99.95%, and 99.89%, respectively [25]. All the above results demonstrated that NIPT has high sensitivity and specificity and can effectively prevent birth defects. The prevalence of positive NIPT results following euploid embryo implantation is 0.7% [26], and that following natural pregnancy is 1.2% [27]. A follow-up of clinical pregnancy after euploid embryo implantation by using PGT-A revealed that only 0.13% of patients developed chromosomal abnormalities during pregnancy. As mentioned above, CVS or amniocentesis increase the risk of miscarriage [28]. Therefore, NIPT offers patients who undergo PGT additional options for prenatal diagnosis [26–28]. However, because of false-positive or false-negative results, the application of NIPT in PGT is still controversial, and studies on the clinical outcomes of patients who receive NIPT in PGT are rare.

In this study, we first analysed the differences in clinical outcomes between patients who underwent invasive prenatal testing (IPT) or NIPT after achieving pregnancy by PGT. The findings of this study may lead to the use of a new pregnancy diagnostic strategy for patients who undergo PGT.

## Materials and methods

### Study design

**Fig. 1:**
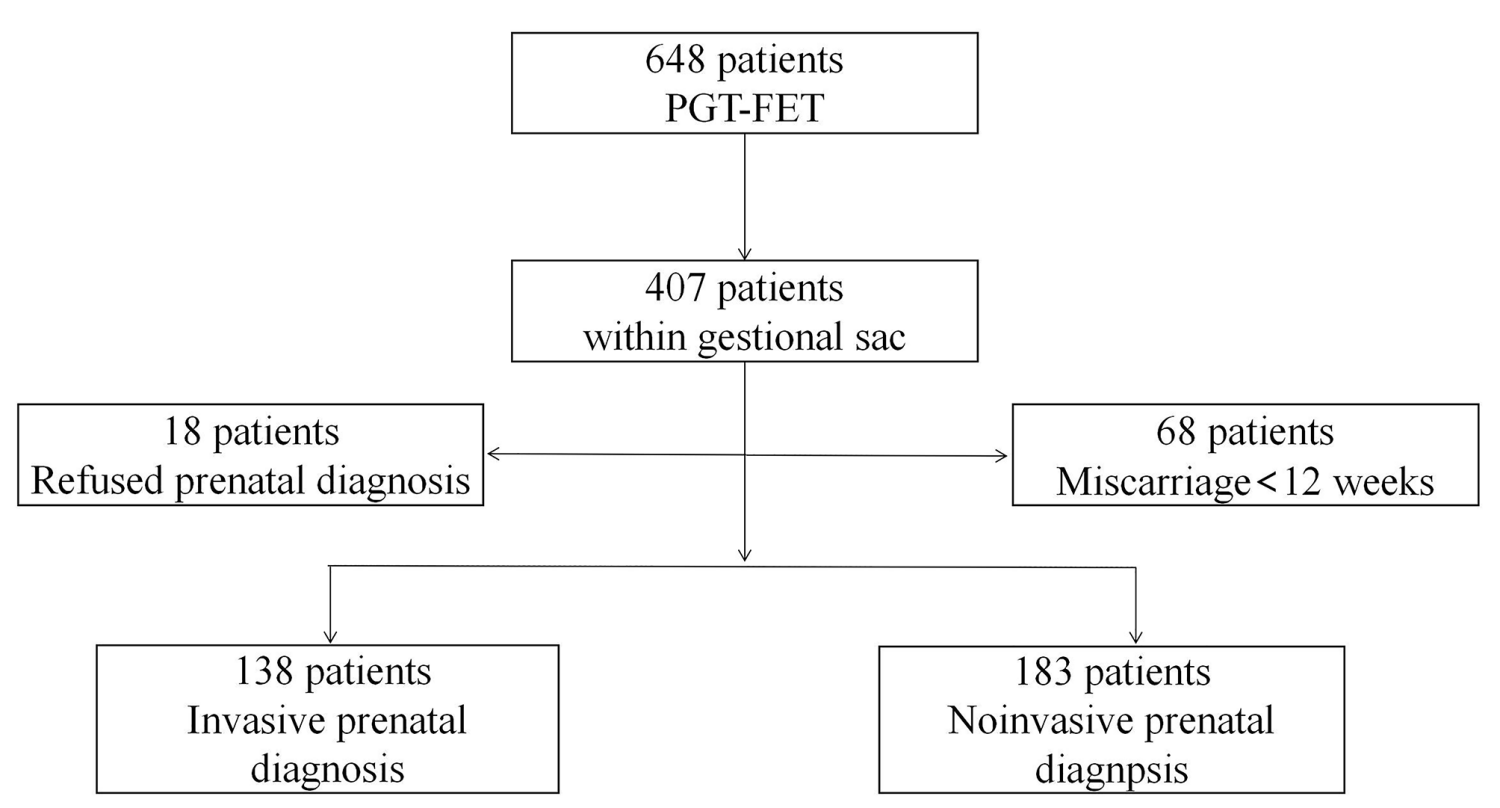
Diagram of study design. Patients grouped according to their pregnancy diagnosis methods

We conducted a retrospective cohort study of patients aged > 20 to 45 years who underwent PGT at our IVF centre between January 2017 and December 2022. Patients who achieved pregnancy after single euploid blastocyst transfer (SET) were required to receive prenatal diagnosis and release related medical records. The patients were divided into 2 groups according to the phase of prenatal diagnosis. Group 1 included patients who accepted IPT (CVS or amniocentesis), and Group 2 included patients who refused CVS or amniocentesis but underwent NIPT. The details of the study design are shown in Fig. 1.

### Patients

Patients who were authorized to provide medical records and agreed to participate in this study were included. Patients who refused to provide medical records or were unwilling to participate in the study were excluded. The following patient clinical information was retrieved from the electronic medical records: age, body mass index (BMI), stimulation duration, total Gn dose, basal follicle-stimulating hormone (FSH), luteinizing hormone (LH), oestradiol (E2), anti-Müllerian hormone (AMH), and PGT indication. Patients within the gestational sac following single blastocyst implantation were eligible for our study.

### Ovarian stimulation and embryo culture

Ovarian stimulation was conducted by using a standard protocol with gonadotropin dose adjustment for the ovarian reserve. Oocyte maturation was triggered when leading follicles were ≥ 17 mm and oocyte retrieval was conducted 36 h later. Intracytoplasmic sperm injection (ICSI) was used for insemination. All the embryos were cultured to the blastocyst stage in 6% CO_2_ and 5% O_2_. All embryos were transferred into G1 medium (Vitrolife, 10,128) from day 1 to day 3 and subsequently transferred into G2 medium (Vitrolife, 10,029) from day 3 to day 6.

### Embryo biopsy and PGT procedures

Blastocyst quality was assessed prior to TE biopsy. Only blastocysts with a quality classification ≥ 3 BB were considered for TE biopsy. All biopsy procedures were performed on the heated stage of a Nikon IX-70 microscope equipped with micromanipulation tools. The detailed procedures have been outlined in a previous report from our group. For TE biopsies, the biopsy products were transferred into microcentrifuge tubes containing 2 ml of PBS and prepared for analysis by multiple displacement amplification (MDA) using an REPLI-g Single-cell Kit (Qiagen). The biopsies were stored at -20 °C for one week if MDA was not detected [14]. Preimplantation genetic testing for aneuploidy and pathogenic genes was performed using the MiSeq NGS platform (Illumina) or Karyomapping platform (Illumina) following the manufacturer’s protocol. The sequencing data of the NGS platform were analysed by Peking Jabrehoo Med Tech Co., Ltd. The data of the Karyomapping platform were analysed by BlueFuse Multi V4.0 (Illumina) [29, 30].

### Embryo transfer and clinical follow-up

Single frozen-thawed embryo transfer (FET) was programmed as previously described [31]. The endometrium was prepared for transfer by hormone replacement treatment (HRT). If the endometrial thickness was ≥ 8, this cycle was considered suitable for FET [32]. Pregnancy was verified by measuring the serum β-hCG concentration 14 days after blastocyst transfer. Clinical pregnancy was defined as observation of a gestational sac with or without a foetal heartbeat on ultrasound evaluation 4 weeks after FET. Clinical miscarriage was defined as failure of the pregnancy failed to progress after detection of an intrauterine gestational sac via pelvic ultrasonography [33]. Live birth was defined as the delivery of a live foetus after 22 weeks of gestation. Preterm birth was defined as a live birth that occurred before 37 completed weeks of pregnancy [34]. Congenital anomalies were defined as structural or functional disorders that occur during intrauterine life and can be identified prenatally, at birth or later in life [35, 36].

### Statistical analysis

Statistical analysis was performed using SPSS 19.0 (SPSS, Inc., Chicago, USA). Continuous variables are presented as the mean ± standard deviation (SD), and Student’s t tests or Mann‒Whitney U tests were conducted to assess statistically significant differences. Categorical variables are expressed as percentages and were analysed using the χ2 or Fisher’s exact test depending on the sample size. P values less than 0.05 were considered to indicate statistical significance.

## Results

### Demographic characteristics of the study population

In total, 648 patients underwent FET after PGT, 407 patients had a single gestational sac confirmed by ultrasound detection, and 321 patients were pregnant. These patients were included in the study and were divided into 2 groups. A total of 138 patients (43.0%) accepted IPT (Group 1), and 183 patients (57.0%) underwent NIPT and refused IPT (Group 2). Among the remaining patients, 18 refused a prenatal diagnosis, and 68 patients who had suffered miscarriage in early pregnancy, which was too early to receive prenatal diagnosis. The demographic data of the 321 patients are shown in Table 1. The patients’ ages varied significantly among the 2 groups (*P* < 0.01). The patients in Group 1 were younger than those in Group 2 (31.04 ± 4.15 vs. 35.64 ± 4.74 years, *P* < 0.001). Among the 2 groups, group 1 had the highest LH (4.30 ± 2.68 vs. 3.40 ± 1.88, *P* = 0.003) and AMH (5.55 ± 11.22 vs. 4.09 ± 3.55, *P* = 0.012) levels. The stimulation durations were also significantly different between the 2 groups (10.61 ± 1.35 vs. 10.19 ± 1.73, *P* = 0.008); however, there were no significant differences in other parameters, such as BMI; FSH, E2, and AMH levels; endometrial thickness; and total Gn dose, between the 2 groups.

**Table 1:** The demgraphic characteristics of 321 PGT-FET cycles

Parameters	Group 1 (*n* = 138)	Group 2 (*n* = 183)	*P*-value
Age (years)	31.04 ± 4.15	35.64 ± 4.74	**< 0.000**
BMI (kg/m^2^)	21.56 ± 3.04	21.94 ± 2.70	0.277
Basal FSH ( IU/L)	6.19 ± 2.15	6.27 ± 2.13	0.525
Basal E2 (pg/ml)	141.03 ± 86.28	157.27 ± 205.46	0.680
Basal LH ( IU/L)	4.30 ± 2.68	3.40 ± 1.88	**0.003**
AMH (ng/mL)	5.55 ± 11.22	4.09 ± 3.55	**0.012**
Endometrial thickness (mm)	9.78 ± 1.49	9.67 ± 1.51	0.600
Stimulation duration (days)	10.61 ± 1.35	10.19 ± 1.73	**0.008**
Total Gn dose (IU)	2363.04 ± 792.94	2412.24 ± 833.61	0.583
β-HCG (IU/L)	920.28 ± 575.19	949.39 ± 747.09	0.837

Values are presented as mean ± standard deviation (SD) or number (%)

Age, age at oocyte retrieval; BMI, body mass index; FSH, follicle-stimulating hormone; E2, estradiol;LH, luteinizing hormone; AMH, anti-Mullerian hormone; Gn, gonadotropin; β-HCG, β-human chorionic gonadotropin level at 12 days after FET.

### Clinical outcomes

The clinical results of each group are shown in Table 2. In group 1, the miscarriage rate was 0.7% (1/138), the live birth rate was 99.3% (137/138), the preterm birth rate was 0.7% (1/138), the birth defect rate was 2.9% (4/138), and the neonatal weight was 3.27 ± 0.42 kg. In group 2, the miscarriage rate was 2.2% (4/183), the live birth rate was 97.8% (179/183), the preterm birth rate was 3.3% (6/183), the birth defect rate was 0.5% (1/183), and the neonatal weight was 3.24 ± 0.56 kg. Clinical outcome comparisons between group 1 and group 2 did not reveal significant differences. Next, we performed comparisons of clinical outcomes between patients who accepted IPT and those who accepted NIPT with identical PGT indications. The results are shown in Table 3. In the PGT-A cohort, the miscarriage rate (0.00 [0/9] vs. 3.3% [5/150], *P* = 1.00), live birth rate (100% [9/9] vs. 96.7% [145/150], *P* = 1.00), preterm birth rate (0.00 [0/9] vs. 0.7% [1/150], *P* = 1.00) and birth defect rate (0.00 [0/9] vs. 0.7% [1/150], *P* = 1.00) of the IPT and NIPT groups were not significantly different. In the PGT-SR cohort, the between-group comparisons of miscarriage rate (1.3% [1/75] vs. 0.0 [0/13], *P* = 1.00), live birth rate (98.7% [74/75] vs. 100.0% [13/13], *P* = 1.00), preterm birth rate (1.3% [1/75] vs. 100.0% [13/13], *P* = 1.00) and birth defect rate (1.3% [1/75] vs. 100.0% [13/13], *P* = 1.00) did not yield significant differences. In the PGT-M cohort, similar results were obtained; specifically, in the IPT and NIPT groups, the miscarriage and preterm birth rates were both 0.0%, and the live birth rates were all 100.0%. The birth defect rates were 5.6% (3/54) and 0.0% (0/20) (*P* = 0.164).

**Table 2:** The IVF clinical outcomes of different prenatal diagnosis groups

Parameters	Group 1 (*n* = 138)	Group 2 (*n* = 183)	*P*-value
Miscarrage, % (n)	0.7 (1/138)	2.2 (4/183)	0.554
Live birth, %(n)	99.3 (137/138)	97.8 (179/183)	0.554
Preterm birth, %(n)	0.7 (1/138)	3.3 (6/183)	0.248
Birth defects, % (n)	2.9 (4/138)	0.5 (1/183)	0.219
Neonate weight (kg)	3.27 ± 0.42	3.24 ± 0.56	0.837

Values are presented as mean ± standard deviation (SD) or number (%)

**Table 3:** The IVF clinical outcomes of different PGT indication groups

Parameters	PGT-A			PGT-SR			PGT-M		
	IPT (*n* = 9)	NIPT (*n* = 150)	*P*-value	IPT (*n* = 75)	NIPT (*n* = 13)	*P*-value	IPT (*n* = 54)	NIPT (*n* = 20)	*P*-value
Miscarriage, % (n)	0	3.3(5/150)	1.00	1.3 (1/75)	0	1.00	0	0	NA
Live birth, %(n)	100.00 (9/9)	96.7 (145/150)	1.00	98.7 (74/75)	100.00 (13/13)	1.00	100.00 (54/54)	100.00 (20/20)	NA
Preterm birth, %(n)	0	0.7 (1/150)	1.00	1.3 (1/75)	0 (0/13)	1.00	0	0	NA
Birth defects, % (n)	0	0.7 (1/150)	1.00	1.3 (1/75)	0 (0/13)	1.00	5.6 (3/54)	0	0.164
Neonate weight (kg)	3.13 ± 0.34	3.23 ± 0.58	0.38	3.33 ± 0.42	3.34 ± 0.50	0.93	3.20 ± 0.44	3.27 ± 0.46	0.96

Values are presented as mean ± standard deviation (SD) or number (%)

NA, not available

## Discussion

In our study, we retrospectively analysed and compared the data of 321 patients who underwent PGT-FET cycles and who accepted IPT (Group 1) or refused IPT but accepted NIPT (Group 2). The demographic data showed that the patients in Group 2 had lower basal LH and AMH levels, and these results may be related to their older age (35.64 ± 4.74 years, *P* < 0.001). These results are similar to those in previous reports, and female age may affect the basal LH and AMH levels of these patients [37]. The duration of ovarian stimulation largely depends on the ovarian response to hormonal stimulation. Age is a critical factor influencing ovarian response in females, so Group 2 had the shortest stimulation duration (10.19 ± 1.73 days, *P* = 0.008) [38]. We performed comparisons of the clinical outcomes between the 2 groups. The miscarriage rate, live birth rate, preterm birth rate, birth defect rate and neonatal weight did not significantly differ between the 2 groups. We also further explored the differences in clinical outcomes between patients who accepted IPT and those who accepted NIPT with identical PGT indications. None of the comparisons yielded significant differences. Although we implanted euploid blastocysts in 321 patients enrolled in our study, 5 cases of birth defects were identified after delivery. There were 4 cases of birth defects in Group 1; 3 were in the PGT-M cohort, and 1 was in the PGT-SR cohort. In Group 2, only 1 case of birth defects was identified, and this case was from the PGT-A cohort. In all 5 cases, the birth defects were limb defects.

In their previous study, Kimelman et al. found that 50 patients (73.5%) opted for non-invasive prenatal screening, 5 (7.4%) underwent invasive testing (4 had CVS and 1 had amniocentesis), and 13 patients (19%) declined further genetic testing [39]. This study demonstrated that most of the patients who underwent PGT-A did not pursue an invasive pregnancy diagnosis and preferred the non-invasive pregnancy diagnosis. In a similar study, Buttle et al. reported that patients who undergo ART are more likely to pursue CVS because of a personal or family history of genetic abnormalities or foetal anomalies than because of a spontaneous pregnancy [40]. The studies above focused on demographic characteristics and involved various groups of patients. Our study system analysed the clinical results of patients who underwent different prenatal diagnosis methods during the PGT cycle. The results showed that there were no significant differences between the two groups.

There are several limitations to our study. First, because this was a retrospective study, the bias regarding demographic characteristics could not be avoided. Second, this study had a relatively small sample size; therefore, to more precisely evaluate reliability of different prenatal diagnostic methods for PGT, further studies with larger sample sizes are needed. Third, NIPT is limited in its ability to detect one or more mutations in several genes simultaneously. This is due mainly to the small amount of cfDNA and foetal-derived fragments that can be obtained and to the deep coverage required [41]. Moreover, the pathogenic gene status of patients in PGT-M cycles cannot be evaluated by NIPT.

In conclusion, the clinical pregnancy outcomes of Groups 1 and 2 are not significantly different. NIPT can be an alternative for PGT-A and PGT-SR. Due to the limitations of current NIPT for monogenic disease, patients who undergo PGT-M are advised to receive amniocentesis or CVS.

## Data Availability

The data that support the findings of this study are available from Guangzhou Women and Children’s Medical Center but restrictions apply to the availability of these data, which were used under license for the current study, and so are not publicly available. Data are however available from the corresponding author upon reasonable request and with permission of Guangzhou Women and Children’s Medical Center.
